# Editorial: Joining efforts to improve data quality and harmonization among European population-based cancer registries

**DOI:** 10.3389/fonc.2024.1496574

**Published:** 2024-10-09

**Authors:** Carmen Martos, Francesco Giusti, Liesbet Van Eycken, Otto Visser

**Affiliations:** ^1^ European Commission, Joint Research Centre (JRC), Ispra, Italy; ^2^ Rare Diseases Research Unit, Foundation for the Promotion of Health and Biomedical Research in the Valencian Region (FISABIO), Valencia, Spain; ^3^ Belgian Cancer Registry, Brussels, Belgium; ^4^ Department of Registration, Netherlands Comprehensive Cancer Organisation (IKNL), Utrecht, Netherlands

**Keywords:** population-based cancer registries, data quality, harmonization, data comparability, Europe

The aim of population-based cancer registries (PBCRs) is to collect information from all new cases of cancer that occur in a defined population (1). They play an essential role in cancer surveillance, quantifying the burden of cancer in terms of incidence, prevalence and survival at population level, describing geographical variation and time trends. In addition, PBCRs are an important information source for planning and evaluating cancer control policies and healthcare systems (2, 3).

The reliability, use and comparability of the data provided by PBCRs depend on their quality as well as the harmonization of data collection and processing, coding and case definition.

The aim of this Research Topic was to share experiences on cancer data quality and harmonization in Europe, focusing on: 1) challenges in data comparability among PBCRs; 2) description of tools and activities for improving cancer data quality and harmonization; 3) Assessment of data quality in PBCRs; 4) challenges in data quality and harmonization related to national data protection regulations; 5) impact of data quality and harmonization on cancer indicators; and 6) epidemiological and statistical methods for improving data comparability.

Three of the fifteen articles included in the Research Topic focus on tools for checking internal consistency of cancer registry data. Giusti et al. give an overview of the Joint Research Centre-European Network of Cancer Registries Quality Check Software (JRC-ENCR QCS), describing its role in processing data files submitted by PBCRs contributing to the European Cancer Information System (ECIS) and its functionalities. The JRC-ENCR QCS is a Java standalone desktop software developed and updated by the JRC to support the validation of cancer registry data. It can be freely downloaded from the ENCR website (4).

Tagliabue et al. compared the functional features and the output differences between the JRC-ENCR QCS and the IARC/IACR CHECK program developed by the International Agency for Research on Cancer (IARC).

Nicholson et al. presented the design of an ontology approach to model the ENCR rules (5) for validating childhood tumors, including some examples of how the ontology handles the ENCR data-validation requirements.

Indicators related to the four dimensions of data quality have been used to evaluate PBCR data: completeness, validity, comparability and timeliness (6, 7). The article “*Quality indicators: completeness, validity and timeliness of cancer registry data contributing to the European Cancer Information System*” Giusti et al. reported the quality indicators from 130 European PBCRs and their time trends using the data collected in the 2015 ENCR-JRC data call. The results provided by this paper could be used as the baseline for monitoring PBCRs data quality indicators in Europe over time.

Two articles by Galceran et al. and by Visser et al. included the current ENCR Recommendations for recording/reporting urothelial tumors and the ENCR Recommendations for coding the basis of diagnosis, respectively. The ENCR Recommendations (8) provide common definitions and rules to improve the data comparability among European PBCRs.

The role of the PBCR in cancer surveillance in term of incidence is shown in two papers by (Giusti et al.) and (Trallero et al.). The article by Giusti et al. highlights geographical and time trend differences in esophageal and gastric cancer in Europe by sub-sites and morphology subgroups. A wide variability in oesophago-gastric cancers was observed, with a corresponding improvement in accuracy of registration in the analyzed period. Trallero et al. described the incidence of hematological malignancies among children in Spain during the period 1983-2018 and compared their results with other Southern European countries. Main diagnostic sub-groups of the International Classification of Childhood Cancer (2017 update) were used for reporting their results.

Three papers focused on prevalence methodology. Demuru et al. explored the validity of alternative versus standard completeness indexes for estimating complete cancer prevalence in Europe. Toffolutti et al. described the procedures to derive complete prevalence and some indicators of cancer cure using data provided by Italian PBCRs. Francisci et al. proposed a new method for estimating short term projections on cancer prevalence by phase of care (initial, continuing and final) that applies to geographical areas covered by cancer registration.

Technological advances and record linkage have contributed to the improvement of the data provided by the PBCR (9, 10). Stage and treatment variables are recommended by the ENCR to be recorded in the European PBCRs (11).

The article by Giusti et al. gives an overview of reporting and using cancer treatment data provided by the European PBCRs. A literature review, conference proceedings and data from 125 European cancer registries contributing to the 2015 ENCR-JRC data call were used to explore the current situation of cancer treatment registration in Europe.

Lopez-Cortes et al. reported the experiences of the International Benchmarking of Childhood Cancer Survival by Stage (BENCHISTA) project to ensure data quality, harmonization and comparability among the CRs participating in the project.

The application of the European General Data Protection Regulation (GDPR) (12) since 2018 has complicated the sharing of health data among European countries, in particular in the Nordic countries due to a stricter interpretation of the GDPR. Larønningen et al. described a new GDPR-compliant federated analysis programme (nordcan.R) and how to use it for computing statistics for the Nordic cancer statistics web platform NORDCAN. The programming languages used for nordcan.R were R and Stata.

Finally, Giusti et al. highlight the recent and ongoing activities of the ENCR, the JRC and the European PBCRs in data quality and harmonization.

In summary, the fifteen articles (9 original research, 3 technology and code, 2 method and 1 perspective) published on this Research Topic provide an overview of the efforts and collaborations among European PBCRs, stakeholders, the ENCR and the JRC to improve data quality and harmonization of European cancer registries. This will contribute to the knowledge of cancer epidemiology in Europe and improve insights in cancer inequalities among European countries and regions. In addition, the Research Topic “Joining Efforts to Improve Data Quality and Harmonization Among European Population-Based Cancer Registries” could provide some important elements for the current Joint Action EU4H-2024-JA-IBA-03, direct grants to Member States’ authorities: to support quality improvement of cancer registry data feeding the European Cancer Information System (13).
